# Ursodeoxycholic acid improves feto-placental and offspring metabolic outcomes in hypercholanemic pregnancy

**DOI:** 10.1038/s41598-020-67301-1

**Published:** 2020-06-25

**Authors:** Luiza Borges Manna, Georgia Papacleovoulou, Flavia Flaviani, Vanessa Pataia, Asaad Qadri, Shadi Abu-Hayyeh, Saraid McIlvride, Eugene Jansen, Peter Dixon, Jennifer Chambers, Marta Vazquez-Lopez, Annika Wahlström, Negusse Kitaba, Hanns-Ulrich Marschall, Keith M. Godfrey, Karen Lillycrop, Catherine Williamson

**Affiliations:** 1 Division of Women and Children’s Health, King’s College London, London, United Kingdom; 2 NIHR Biomedical Research Centre at Guy’s and St Thomas’ Foundation Trust, London, United Kingdom; 3 Centre for Health Protection, National Institute for Public Health and the Environment, Bilthoven, The Netherlands; 4 Women’s Health Research Centre, Surgery and Cancer, Faculty of Medicine, Hammersmith Hospital, Imperial College London London, United Kingdom; 5 Department of Molecular and Clinical Medicine/Wallenberg Laboratory, Sahlgrenska Academy, University of Gothenburg, Gothenburg, Sweden; 6 MRC Lifecourse Epidemiology Unit and NIHR Southampton Biomedical Research Centre, University of Southampton and University Hospital Southampton NHS Foundation Trust, Southampton, United Kingdom; 7 Biological Sciences, University of Southampton, Southampton, United Kingdom

**Keywords:** Metabolic disorders, Translational research

## Abstract

Perturbations in the intrauterine environment can result in lifelong consequences for metabolic health during postnatal life. Intrahepatic cholestasis of pregnancy (ICP) can predispose offspring to metabolic disease in adulthood, likely due to a combination of the effects of increased bile acids, maternal dyslipidemia and deranged maternal and fetal lipid homeostasis. Whereas ursodeoxycholic acid (UDCA) is a commonly used treatment for ICP, no studies have yet addressed whether it can also prevent the metabolic effects of ICP in the offspring and fetoplacental unit. We therefore analyzed the lipid profile of fetal serum from untreated ICP, UDCA-treated ICP and uncomplicated pregnancies and found that UDCA ameliorates ICP-associated fetal dyslipidemia. We then investigated the effects of UDCA in a mouse model of hypercholanemic pregnancy and showed that it induces hepatoprotective mechanisms in the fetal liver, reduces hepatic fatty acid synthase (Fas) expression and improves glucose tolerance in the adult offspring. Finally, we showed that ICP leads to epigenetic changes in pathways of relevance to the offspring phenotype. We therefore conclude that UDCA can be used as an intervention in pregnancy to reduce features of metabolic disease in the offspring of hypercholanemic mothers.

## Introduction

The prevalence of non-communicable diseases such as obesity and type 2 diabetes has grown rapidly over the last three decades^1^. Increasing evidence links gestational, perinatal and early infancy factors to susceptibility of metabolic disease in adulthood^2^. Type 2 diabetes is more prevalent among people whose mothers experienced malnutrition during the Dutch famine in 1944-45^3^, and a substantive literature suggests that maternal obesity and gestational diabetes mellitus (GDM) are associated with elevated metabolic syndrome markers in childhood and later life^4^. These observational studies have been strengthened by studies in animal models, which have shown that the maternal nutritional and metabolic status can profoundly influence cardiovascular and metabolic function in young and adult offspring^5–7^.

Intrahepatic cholestasis of pregnancy (ICP) affects 0.5% of pregnancies in UK and up to 2% globally^8^. Its etiology is complex, with genetic and endocrine components. A subgroup of affected women possess genetic variants in biliary transporters (*ABCB4/MDR3* and *ABCB11/BSEP*) and nuclear receptors (*NR1H4/FXR*) that perturb bile acid (BA) homeostasis^9,10^. The phenotype of ICP is unmasked by pathologically elevated levels of reproductive hormone metabolites in genetically predisposed women^11–14^. Women with ICP present with pruritus, hepatobiliary injury and hypercholanemia (elevated serum BA concentrations), as well as dyslipidemia^2,3,15^. Increased rates of GDM have been reported^15,16^. Maternal BA concentrations ≥40 μmol/L are associated with adverse fetal outcomes, including spontaneous preterm labor, fetal hypoxia and meconium-stained amniotic fluid^17–19^. Fetal BA levels are raised in umbilical cord blood in ICP pregnancies^20^.

Exposure of the fetus to elevated BA *in utero* as a result of ICP predisposes the adolescent offspring to metabolic disease. Analysis of the Northern Finland Birth cohort database (1986) revealed that 16-year old children from cholestatic pregnancies had increased body mass index and dyslipidemia, and the males had raised fasting insulin compared to age matched adolescents of uncomplicated pregnancies^21^. In a mouse model of 0.5% cholic acid (CA) feeding that replicates the hypercholanemia of cholestatic pregnancy, female offspring exposed to increased BAs *in utero* developed hepatic steatosis, glucose intolerance and insulin resistance when challenged with a Western diet (WD) for six weeks. Human samples and *in vivo* experiments showed that increased transplacental cholesterol transport and greater fat storage in the fetoplacental unit contribute to this phenotype^21^. Furthermore, maternal hypercholanemia resulted in altered DNA methylation patterns in the agouti *Avy* yellow mouse offspring, indicating epigenetic alterations in the ICP-associated offspring phenotype^21^.

Administration of ursodeoxycholic acid (UDCA) is a treatment for ICP that reduces BA levels and improves symptoms and biochemical abnormalities in some mothers. However, it is not known whether UDCA improves fetal abnormalities and susceptibility of the offspring to metabolic disease later in life^22,23^.

We hypothesized that treatment with UDCA in cholestatic pregnancy can reverse the fetal metabolic phenotype, as well as placental dyslipidemia. This may also prevent features of metabolic syndrome in the adult offspring. To address our hypothesis, we first compared the fetal and maternal biochemical profile in UDCA-treated ICP pregnancies, untreated ICP and uncomplicated pregnancies. Based on these findings, we then administered UDCA in a mouse model of maternal hypercholanemia to mimic UDCA treatment in ICP^21^ and we investigated the impact on the metabolic phenotype of the adult offspring. Based on our previous findings in the agouti *Avy* yellow mouse models and the emerging evidence that epigenetic processes in early life stages are key factors that influence metabolism^24,25^, we also explored modifications in the methylation patterns of umbilical cord leukocytes and placentas from human ICP using an epigenome-wide association study (EWAS) and global methylation studies.

## Methods

### Human samples

Women with untreated ICP (n = 16), UDCA-treated ICP (n = 17) and controls (n = 18) were recruited from Queen Charlotte’s and Chelsea and St Thomas’ Hospitals, London. ICP diagnosis was based on presentation with gestational pruritus and serum BA over 10 µmol/L and no additional identifiable cause of liver dysfunction. Exclusion criteria included other causes of hepatic dysfunction (including pre-eclampsia, hemolysis, elevated liver enzymes, and low platelets (HELLP) syndrome, acute fatty liver of pregnancy, primary biliary cholangitis, active viral hepatitis, and any ultrasound abnormality that may result in biliary obstruction) and multi-fetal pregnancy. Controls were women with uncomplicated pregnancies. The decision to treat with UDCA was made by the individual specialist clinician looking after each woman with ICP. Maternal serum and umbilical cord blood were collected and prepared as previously described^20^.

### Animal handling and maintenance

Age-matched (6–8-week-old) female and male C57BL/6 inbred mice were purchased from Harlan Laboratories (Derbyshire, UK) and maintained in a 24-hour light-dark cycle (12 h/12 h) with free access to normal chow diet (RM3; SDS, Essex, UK) and water. Mice were allowed to acclimatize for one week before any experiment. For all studies, the second litters were used as previously described^21^. A week before mating for the second litter, females were randomly assigned to one of the three dietary groups: RM3 control diet (normal chow-NC; n = 6), RM3 diet supplemented with 0.5% CA (CA diet; n = 6) or RM3 diet supplemented with 0.5% CA plus 0.5% UDCA (CA + UDCA diet; n = 4). All diets were purchased from LBS Serving Biotechnology Ltd, Horley, UK. To address the effects of UDCA in gestational cholestasis, pregnant mothers were sacrificed on day 18 of pregnancy after a 4-hour fast. Maternal and fetal liver and blood as well as placentas were collected. Maternal body weight, liver weight, litter number, fetal weight and placental weight were monitored.

To investigate the effects of UDCA on long-term health of the offspring, a different set of mice were placed on an identical maternal dietary regime and were allowed to give birth to offspring (n = 4–6 mothers per dietary regimen). Litters were reduced to four mice per mother on post-natal day 2; 2 females and 2 males from each mother were kept when possible. Only female offspring were analyzed in this study, as it was previously shown that females from this dietary regime develop a more extreme phenotype^21^. At 12 weeks of age, one female mouse from each litter was challenged with a western diet for six weeks (LBS Serving Biotechnology Ltd), whereas female littermates continued on a NC diet(n = 4–6 per dietary regimen from each different diet mothers). A glucose tolerance test was performed two days before sacrifice of the animals at 18 weeks of life as previously described (intraperitoneal injection, 2 g/kg)^21^.

### Biochemical measurements

Serum and tissue biochemical parameters (total, LDL- and HDL-cholesterol, triglycerides (TG) and FFA) were measured using an LX20 autoanalyzer (Beckman Coulter) as previously described^21^. BA measurements were performed by ultra-performance liquid chromatography-tandem mass spectrometry (UPLC-MS/MS) with the use of internal standards for extraction and quantification as previously reported^11^. The same techniques were used for both human and animal studies.

### Epigenome wide association studies(EWAS)

Venous umbilical cord samples were collected from pregnancies affected by ICP (n = 43; 22 of which received treatment with UDCA) and uncomplicated pregnancies (n = 17). Samples were collected in plain vacutainers and centrifuged at 3500 rpm for 10 minutes. The serum was stored at −80 °C until use. EDTA blood collection tubes were used to store whole blood at −80 °C. DNA was extracted using the Qiagen QIAamp Blood mini kit, according to the manufacturers instructions (Qiagen, Skelton House, Manchester, UK). Following elution of DNA into AE buffer, samples were returned to storage at −80 °C until use. 1 µg of the genomic DNA was treated with Sodium Bisulfite using Zymo EZ DNA Methylation-Gold kit (ZymoResearch, Irvine, California, USA, D5007) and processing of the HumanMethylationEPIC (Illumina, Inc. CA, USA) platform was carried out by the Centre for Molecular Medicine and Therapeutics (CMMT) (http://www.cmmt.ubc.ca).

Raw image array files were processed using the R statistical software (R version 3.5.0). A total of 866,836 probes for 60 samples were analysed with minfi (version 1.26) and probe signals normalised using the stratified quantile normalisation algorithm^26^. We identified probes that were detected (P > 0.01) in one or more samples (n = 863,110) and were not at the sites of single-nucleotide polymorphisms (SNPs) (minor allele frequency – maf = 0)^26^ (n = 833,907) or cross-reactive (identified for the EPIC dataset^27^). After excluding probes that did not meet all these criteria, a total of 792,068 probes, including those on sex chromosomes, were maintained for subsequent analyses.

Statistical analyses for the methylation data were performed using the logit transformed M-values for methylation to avoid bias that would have incurred if beta values were selected^28^. Differentially methylated CpGs (dmCpGs) were assigned using Limma (version 3.36.1)^29^ and adjusted for status, gender and ethnicity as confounders as well as treatment, gender and ethnicity in the second model. A 5% Benjamini-Hochberg False Discovery Rate (FDR)^30^ correction for multiple testing was applied. All dmCpGs were assigned to their corresponding genomic context and location using the R/Bioconductor package IlluminaHumaMethylationEPICanno.ilm10b2.hg19 (version 0.6.0)^31^.

Top 1000 dmCpGs, with a p value <1.74E-04 and FDR < 13%, were selected from the disease (treated and untreated) vs control probe-wise differential methylation analyses to perform IPA pathway analyses (QIAGEN Inc., version 01–07). The increase in FDR was adjusted to maintain a significant number of CpGs^32^, including 18 passing FDR at 5%.

Differentially methylated regions were identified using the DMRcate package (version 1.16.0)^33^. Gene ontology (GO) and Kyoto Encyclopedia of Genes and Genomes (KEGG) analyses were performed with R/Bioconductor package missMethyl (version 1.14.0) using the gometh function^34^ and the MethylationEPIC genes as background. Further pathway analyses were completed with Ingenuity IPA (QIAGEN Inc., version 01–07) utilising the Ingenuity knowledge base reference gene set. An IPA search of individual genes was also run to establish their biological significance.

### Global DNA methylation studies

DNA extraction of placentas was carried out with a DNeasy Blood and Tissue Kit (Qiagen) as per supplier’s instructions. DNA was eluted in buffer AE, which guarantees optimal DNA stability, and stored at −20 °C for further use. Quantification of DNA methylation was performed using the Epigentek MethylFlash Methylated DNA Kit (colorimetric assay; Insight Biotechnology Ltd, Wembley, UK), according to manufacturer’s instructions. 200 ng of sample DNA as well as positive and negative controls were included. The light absorbance was quantified in a microplate reader at 450 nm (Pherastar FS, BMG Labtech, Bucks, UK). Final amounts were calculated as percentages based on the controls’ standard curve as instructed by the manufacturer.

### Quantitative real-time PCR

1 μg of total RNA (Qiagen, Manchester UK) from mouse tissues and human placentas was processed as previously described^21^. Primer sequences (Sigma-Aldrich, Poole, UK) are given in Supplementary Table 1.

### Statistical analysis

Data are presented as means ± sem unless otherwise stated. Statistical analysis for multiple comparisons was performed by repeated measures of ANOVA and Newman–Keuls *post hoc* testing with the GraphPad Prism 7.00 software (GraphPad Software Inc., San Diego, CA, USA). The significance cutoff was set at p ≤ 0.05.

### Study approval

Written informed consent was received from participants prior to inclusion in the study, which conformed to the 1975 Declaration of Helsinki guidelines. Biochemical studies were approved by the local ethics committee of Hammersmith Hospital NHS Trust (11/L0/0396). Epigenome-wide studies were also approved by the ethics committees of Hammersmith Hospitals NHS Trust, London (97/5197 and 08/H0707/21), and King’s College Hospitals NHS Trust, London (03WH06).

All experimental procedures were approved by the ethical committee for animal welfare at King’s College London, and all animal studies were compliant with the UK Animals (Scientific Procedures) Act of 1986 and the guidelines from the biological sciences unit at King’s College London.

## Results

### UDCA improves fetal dyslipidemic features associated with ICP

**Figure 1:**
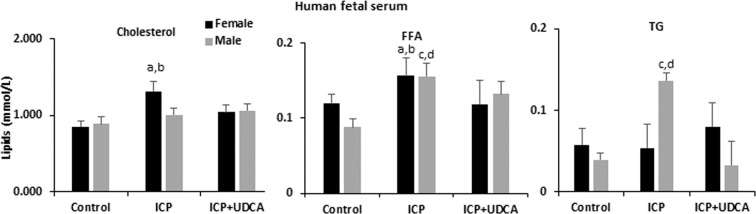
Serum biochemical profile of umbilical cord blood from uncomplicated pregnancies, untreated ICP and UDCA-treated ICP. n = 18, 16 and 17, respectively. Data are presented as mean and standard error of the mean (SEM) and were analyzed with multiple measures of ANOVA followed by Neuman Keul’s post-hoc testing. (a) p < 0.05 for comparison ICP vs Control in females; (b) p < 0.05 for comparison ICP vs ICP + UDCA; (c) p < 0.05 for comparison ICP vs Control in males; (d) p < 0.05 for comparison ICP vs ICP + UDCA males; ICP: intrahepatic cholestasis of pregnancy; UDCA: ursodeoxycholic acid; FFA: free fatty acids; TG: triglycerides.

ICP is associated with umbilical cord blood dyslipidemia and metabolic syndrome in 16-year-old children^21^. Analysis of umbilical cord blood lipid profiles in fetal cord blood from ICP + UDCA treatment showed an increase in cholesterol and FFA in females exposed to ICP, whereas FFA and TG were increased in exposed males. These features were reversed by UDCA treatment (Fig. 1).

### UDCA improves elevated maternal BA concentrations associated with CA feeding and induces fetal hepatoprotective mechanisms in mouse hypercholanemic pregnancy

**Figure 2:**
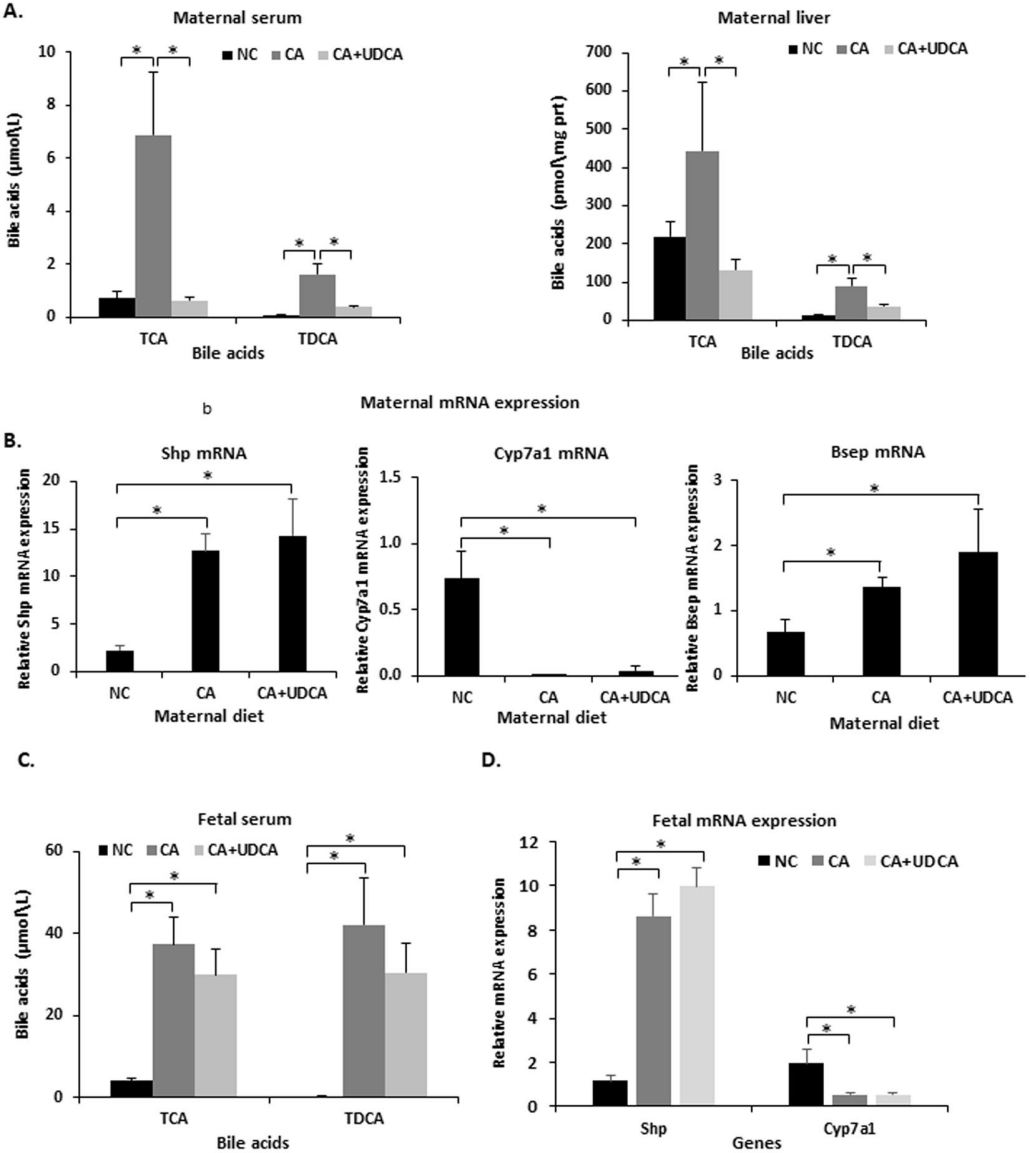
Effects of UDCA on mouse bile acid homeostasis. (**A**) UDCA supplementation in 0.5% CA-fed mice reversed increased taurine-conjugated bile acids in the maternal serum and liver. (**B**) UDCA did not affect maternal hepatic mRNA expression levels of Fxr target genes. (**C**) UDCA supplementation in CA feeding did not affect taurine-conjugated bile acids in the fetal serum (**D**) UDCA supplementation in CA feeding did not affect Shp or Cyp7a1 mRNA expression levels in the fetal liver. Data are presented as mean and standard error of the mean (SEM) and were analyzed with multiple measures of ANOVA followed by Neuman Keul’s post-hoc testing. *p < 0.05 between comparisons connected by lines; NC n = 6, C A n = 6, CA + UDCA n = 4. NC, CA and CA + UDCA represent maternal diet.

Due to the positive effects of UDCA on ICP-induced fetal dyslipidemia and the limited options for *in vivo* human fetal studies in ICP, we expanded our studies into a mouse model in which a 0.5% (CA) diet fed to pregnant mice mimicked the hypercholanemia of ICP, as previously described^21^. We first investigated whether dietary supplementation with UDCA is sufficient to reverse the CA diet-induced increase in maternal and fetal BA concentrations (CA + UDCA group). UDCA prevented the elevation of taurocholic acid (TCA) and taurodeoxycholic acid (TDCA) in maternal serum and liver (Fig. 2A). However, UDCA did not affect the altered mRNA expression of CA diet-induced Fxr targets such as Shp, Cyp7a1 and Bsep (Fig. 2B). Unlike maternal BA levels, UDCA did not significantly reverse the CA diet-induced increase in fetal serum BAs (Fig. 2C). UDCA did not alter fetal hepatic Cyp7a1 and Shp mRNA expression (Fig. 2D).

**Figure 3:**
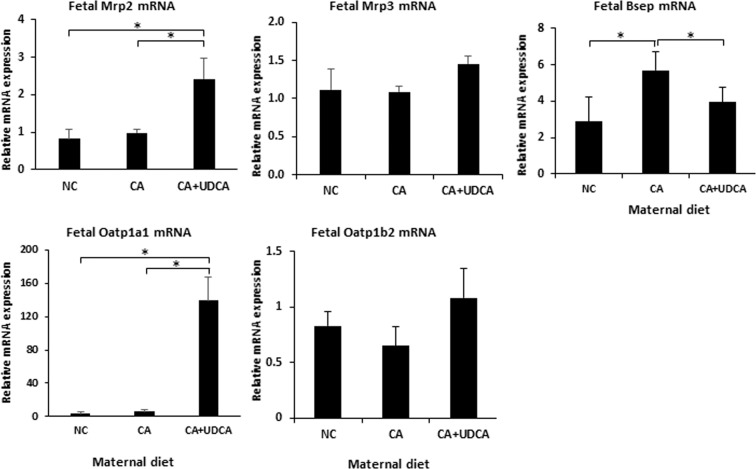
Effects of UDCA on fetal hepatic bile acid transporters. Gene expression profile of the BA transport genes Mrp2, Mrp3, Bsep, Oatp1a1 and Oatp1b2 mRNA in mouse fetal liver. Data are presented as mean and standard error of the mean (sem) and were analyzed with multiple measures of ANOVA followed by Neuman Keul’s post-hoc testing. *p < 0.05 between comparisons connected by lines, NC n = 6, CA n = 6, CA + UDCA n = 4. NC, CA and CA + UDCA represent maternal diet.

As UDCA did not alter CA diet-induced serum BAs and hepatic Fxr targets in the fetus, we investigated whether UDCA can induce fetal hepatoprotective mechanisms. To address this, we studied the gene expression profile of BA transporters in the fetal liver affected by hypercholanemia. UDCA increased Mrp2 and Oatp1a1 mRNA, whereas it reversed the Bsep mRNA upregulation caused by gestational hypercholanemia in the fetal liver (Fig. 3). No statistically significant changes were observed in gene expression of fetal hepatic Oatp1b2 and Mrp3 mRNA.

We previously showed that maternal hypercholanemia in the mouse increased liver to body weight ratio, as well as placental weight. UDCA reversed the increase in placental weight, but it did not affect the maternal liver to body weight ratio (Supplementary Figure 1).

### UDCA improves hypercholanemia-induced maternal and fetal dyslipidemia in the mouse

**Figure 4:**
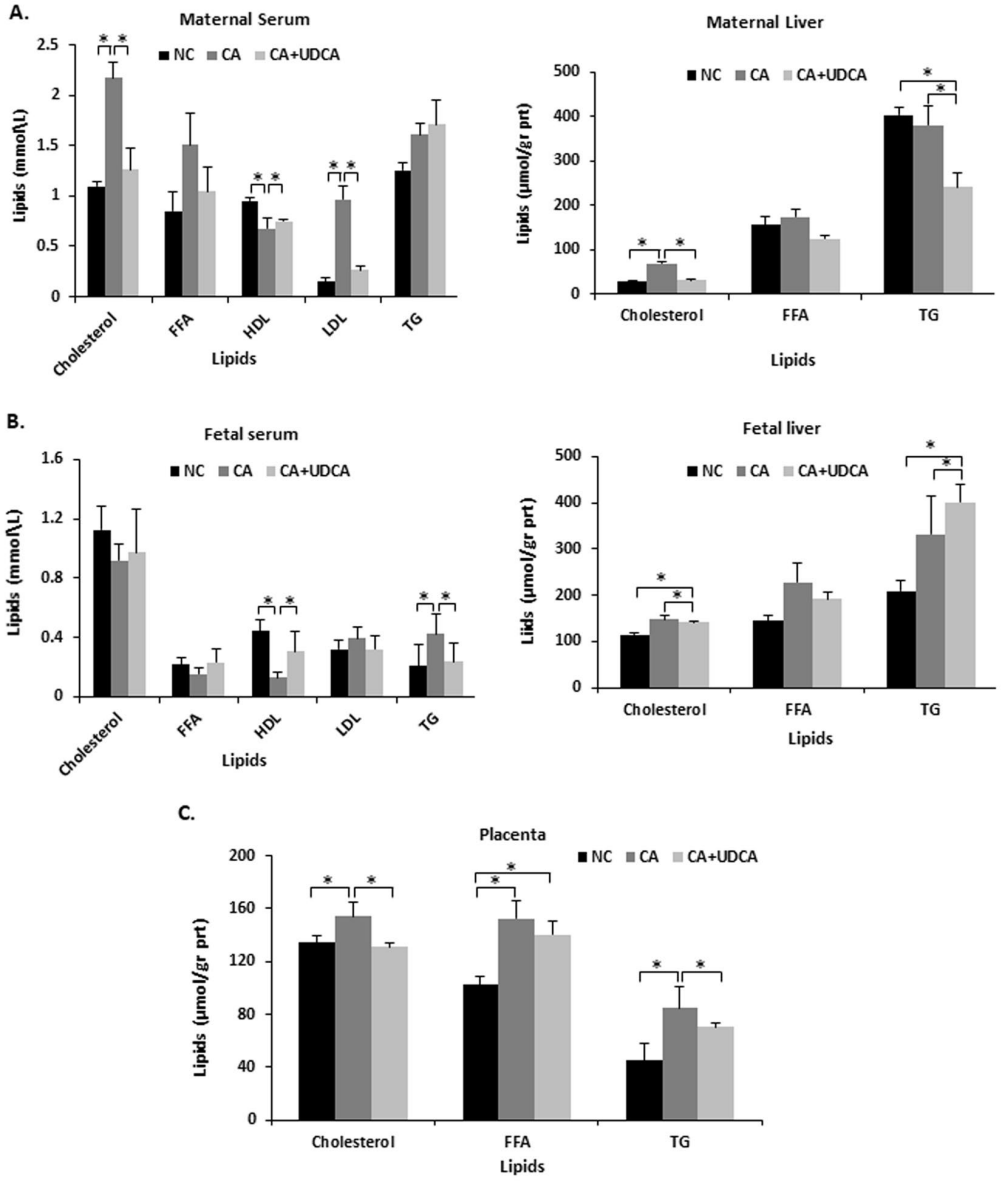
Effects of UDCA on lipid profile in mouse hypercholanemic pregnancy. (**A**) UDCA improved maternal serum cholesterol and LDL-cholesterol as well as hepatic cholesterol increase in gestational cholestasis. (**B**) UDCA reversed cholestasis induced changes in HDL and TG in the fetal serum, but it did not affect cholesterol and TG raised levels in the fetal liver. (**C**) UDCA reversed cholesterol and TG elevated levels associated with maternal cholestasis in placenta. Data are presented as mean and standard error of the mean (sem) and were analyzed with multiple measures of ANOVA followed by Neuman Keul’s post-hoc testing. *p < 0.05 between comparisons connected by lines. NC n = 6, CA n = 6, CA + UDCA n = 4. NC, CA and CA + UDCA represent maternal diet. FFA: free fatty acids; HDL: high-density lipoprotein; LDL: low-density lipoprotein; TG: triglyceride.

We previously established that, in line with ICP, mouse hypercholanemic pregnancy results in maternal and fetoplacental dyslipidemia^15,21^. As shown in Fig. 4A, UDCA prevented the CA diet-induced changes in total, HDL- and LDL-cholesterol in maternal serum. It also reduced hepatic TG and cholesterol levels. In the fetal serum, UDCA reversed the decrease in HDL cholesterol and the increase in TG levels, (Fig. 4B). Also, UDCA supplementation in hypercholanemic pregnancy reversed the increase of cholesterol and TG levels in the placenta (Fig. 4C). However, UDCA did not affect the dyslipidemic phenotype of the fetal liver (Fig. 4B).

**Figure 5:**
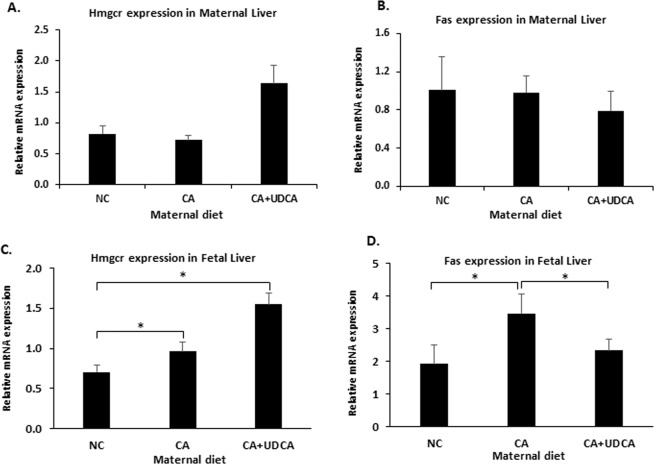
Effects of UDCA on lipid gene expression profile in mouse hypercholanemic pregnancy. (**A**) Hmgcr mRNA expression in maternal liver. (**B**) Fas mRNA expression in maternal liver (**C**) Hmgcr mRNA expression in fetal liver (**D**) Fas mRNA expression in fetal liver. Data are presented as mean and standard error of the mean (sem) and were analyzed with multiple measures of ANOVA followed by Neuman Keul’s post-hoc testing. NC n = 6, CA n = 6, CA + UDCA n = 4. *p < 0.05 between comparisons connected by lines. NC: normal chow; CA: diet supplemented with cholic acid; CA + UDCA: diet supplemented with cholic acid + ursodeoxycholic acid; Hmcr: HMG-coA reductase; Fas: fatty acid synthase.

To further investigate whether maternal and fetal serum lipid profiles are associated with changes in hepatic metabolism, we looked at the transcription of hepatic rate-limiting cholesterol and fatty acid synthesis genes, HMG-CoA reductase (Hmgcr) and fatty acid synthase (Fas) mRNA. No effects were observed in the hepatic Hmgcr mRNA either in the mother or fetus in response to UDCA (Fig. 5A,C). In contrast, UDCA prevented the CA-associated increase of Fas mRNA in the fetal liver (Fig. 5D) but not in the maternal liver (Fig. 5B).

### UDCA in mouse hypercholanemic pregnancy improves glucose tolerance in female offspring

**Figure 6:**
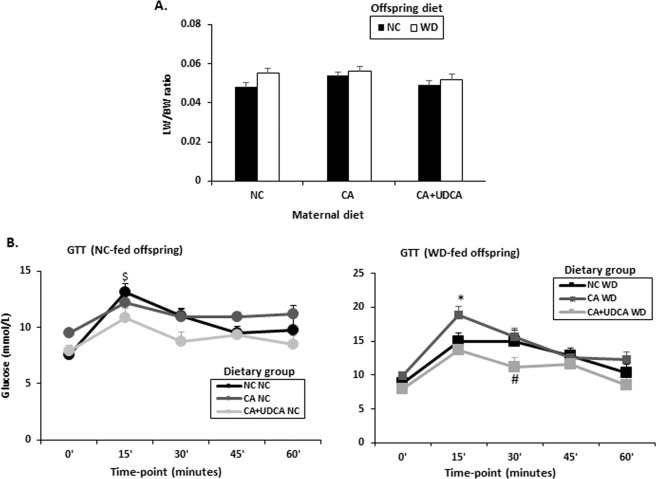
Effects of UDCA on liver to body weight ratio and glucose tolerance in offspring of mouse hypercholanemic pregnancy. (**A**) Effects of UDCA on liver to body weight ratio (**B**) Effects of UDCA on glucose tolerance in NC-fed and WD-fed female offspring of maternal hypercholanemia. Data are presented as mean and standard error of the mean (sem) and were analyzed with multiple measures of ANOVA followed by Neuman Keul’s post-hoc testing. ^$^p < 0.05 0′ vs 15′, *p < 0.05 15′ CA WD vs 15′ NC/CA + UDCA WD, ^#^p < 0.05 30′ CA WD vs 30′ NC/CA + UDCA WD, NC n = 6, CA n = 6, CA + UDCA n = 4. NC/CA/CA + UDCA represent maternal diet; NC/WD represent offspring diet. LW/BW: liver weight/body weight; NC: normal chow; WD: western diet; GTT: glucose tolerance test.

We previously established that female adult offspring exposed to CA *in utero* develop glucose intolerance when fed a WD for six weeks^21^. We therefore tested whether exposure of the offspring to UDCA in pregnancy can ameliorate this phenotype. Although UDCA did not affect the liver to body weight ratio (Fig. 6A), it did significantly improve glucose tolerance in 18-week old female offspring (Fig. 6B). Remarkably, whereas offspring fed a NC diet responded to intraperitoneal glucose injection within 15′ regardless of the maternal dietary regime during pregnancy (Fig. 6B, left panel), offspring fed a WD diet who were exposed to UDCA *in utero* did not develop glucose intolerance (Fig. 6B, right panel). Intriguingly, 30′ after the intraperitoneal glucose injection, blood glucose levels were significantly decreased in WD-fed females exposed to UDCA in gestation compared to WD-fed females from NC- or CA-fed mothers (Fig. 6B).

### ICP alters DNA methylation profiles of relevance to offspring phenotype

Eighteen CpGs were differentially methylated (with a 5% Benjamini-Hochberg FDR) when all cases of ICP (treated and untreated, labelled as disease group) were compared to controls (control versus disease comparison, Table 1). Specifically, these dmCpGs were located mainly in the gene body (TEX29, TBX1, MAMDC2, WDFY4, AHNAK, NFIA and WDR70); within the 5′ untranslated region, between the TSS and the ATG start site (H6PD, ILR21, DFNB59) and one was located in the 3′UTR, between the stop codon and poly A signal (PYCR2) (Table 1). An IPA search of the genes associated with the DMCpGs was then performed to analyse their biological significance. H6PD is related to carbohydrate metabolism biological processes, whilst AHNAK is related to impaired glucose tolerance. When analyses were run to include the treatment as a factor in the model, both the control versus untreated and also the treated versus untreated comparisons identified two significantly differentially methylated CpGs, all assigned to the PRR25 gene (located within the CpG island, Table 1), which does not have any known direct relevance to the phenotype.

**Table 1:** Differentially methylated CpGs and assigned genes in different comparisons. Statistical analysis was performed using the logit transformed M-values for methylation. Differentially methylated CpGs were assigned using Limma (version 3.36.1) and adjusted for status, gender and ethnicity as confounders. A 5% Benjamini-Hochberg False Discovery Rate (FDR) correction for multiple testing was applied. All dmCpGs were assigned to their corresponding genomic context and location using the R/Bioconductor package IlluminaHumaMethylationEPICanno.ilm10b2.hg19 (version 0.6.0). ICP: intrahepatic cholestasis of pregnancy; logFC: log fold change; UCSC: University of California, Santa Cruz genome browser.

Comparison	CpG site	Status	logFC	P.Value	Adjusted P value	Symbol	UCSC Reference Gene Name
Control versus disease (treated and untreated ICP)	cg13274534	Hypermethylated in disease	−0.243564	4.64E-08	0.02264796	TEX29	testis expressed 29
	cg00720629	Hypermethylated in disease	−0.3728101	1.89E-07	0.02264796	TBX1	T- box 1
	cg09781936	Hypermethylated in disease	−0.2377453	2.72E-07	0.02264796	TBX1	T-box 1
	cg18351741	Hypermethylated in disease	−0.3198715	3.09E-07	0.02264796	DFNB59	deafness, autosomal recessive 59
	cg10034679	Hypermethylated in disease	−0.247224	3.19E-07	0.02264796	WDFY4	WDFY family member 4
	cg01520190	Hypermethylated in disease	−0.2302545	3.80E-07	0.02264796	AHNAK	AHNAK nucleoprotein
	cg17676618	Hypermethylated in disease	−0.2464	4.90E-07	0.02544932		
	cg16850951	Hypermethylated in disease	−0.3245504	8.75E-07	0.03848915		
	cg03354554	Hypomethylated in disease	0.47555505	1.79E-07	0.02264796		
	cg23530232	Hypomethylated in disease	0.40697096	2.52E-07	0.02264796	H6PD	hexose-6-phosphate dehydrogenase/ glucose 1-dehydrogenase
	cg08282819	Hypomethylated in disease	0.40336227	2.59E-07	0.02264796	IL21R	interleukin 21 receptor
	cg05018116	Hypomethylated in disease	0.33289686	2.90E-07	0.02264796	BRD4	bromodomain containing 4
	cg10961050	Hypomethylated in disease	0.33797259	3.01E-07	0.02264796	MAMDC2	MAM domain containing 2
	cg25975961	Hypomethylated in disease	0.52015521	3.37E-07	0.02264796		
	cg22559497	Hypomethylated in disease	0.43934031	3.75E-07	0.02264796		
	cg20334115	Hypomethylated in disease	0.50811853	4.00E-07	0.02264796	PYCR2	pyrroline-5-carboxylate reductase 2
	cg25001190	Hypomethylated in disease	0.33147151	5.14E-07	0.02544932	NFIA	nuclear factor I A
	cg06313424	Hypomethylated in disease	0.31707252	6.02E-07	0.02803589	WDR70	WD repeat domain 70
Control versus untreated ICP	cg03749207	Hypomethylated in untreated ICP	1.742272	5.82E-09	0.00460946	PRR25	proline rich 25
	cg02407415	Hypomethylated in untreated ICP	1.449036	5.37E-08	0.02125066	PRR25	proline rich 25
Treated versus untreated ICP	cg03749207	Hypomethylated in untreated ICP	1.789117	2.18E-10	0.00017294	PRR25	proline rich 25
	cg02407415	Hypomethylated in untreated ICP	1.425257	8.48E-09	0.00335754	PRR25	proline rich 25

**Figure 7:**
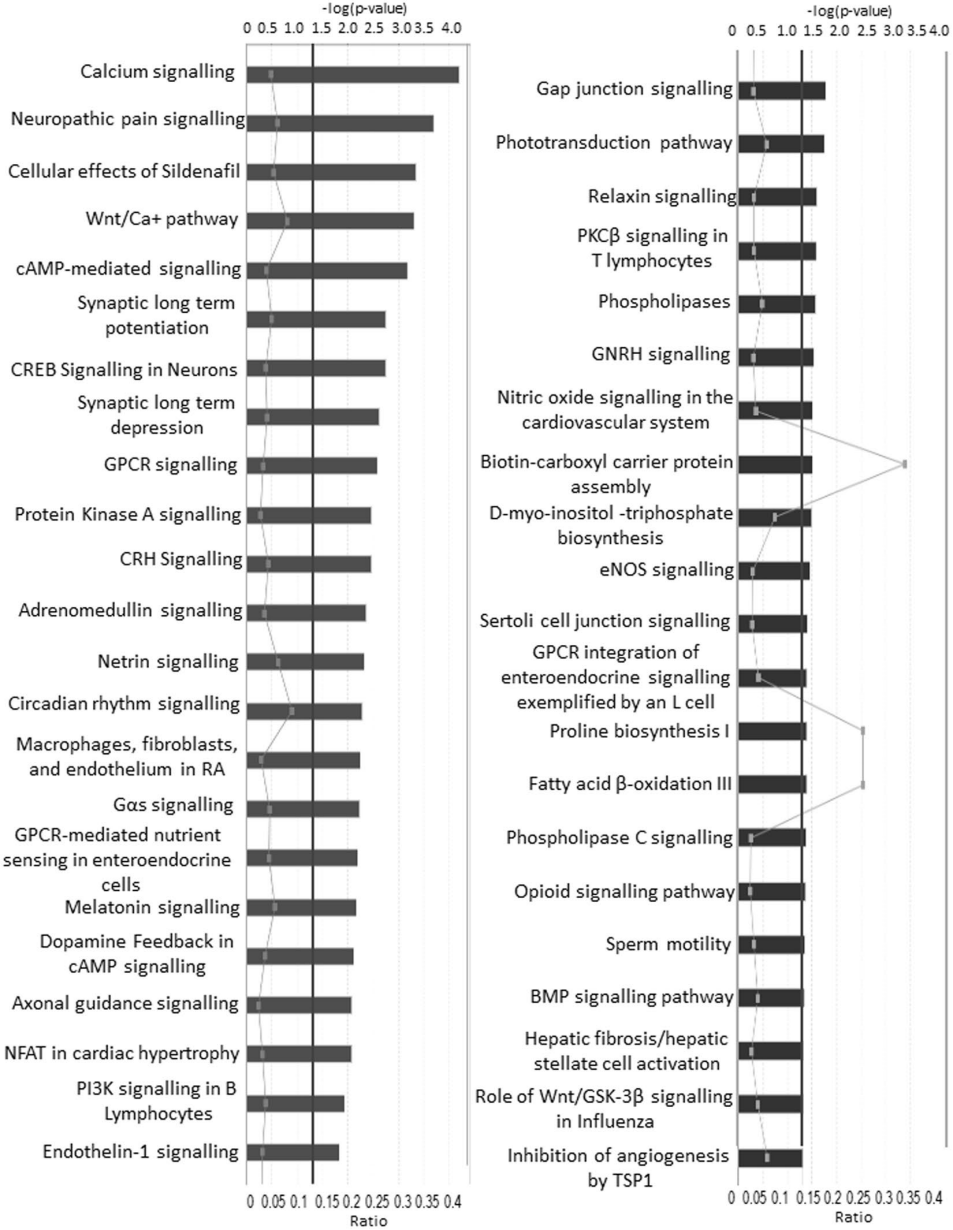
Canonical pathways identified by IPA in the control versus disease comparison (treated and untreated ICP). Bars represent –log (p value). Continuous line represents p value threshold equivalent to 0.05. Lines with squares represent the number of molecules in the dataset that are part of the pathway/number of molecules in the pathway (ratio).

**Figure 8:**
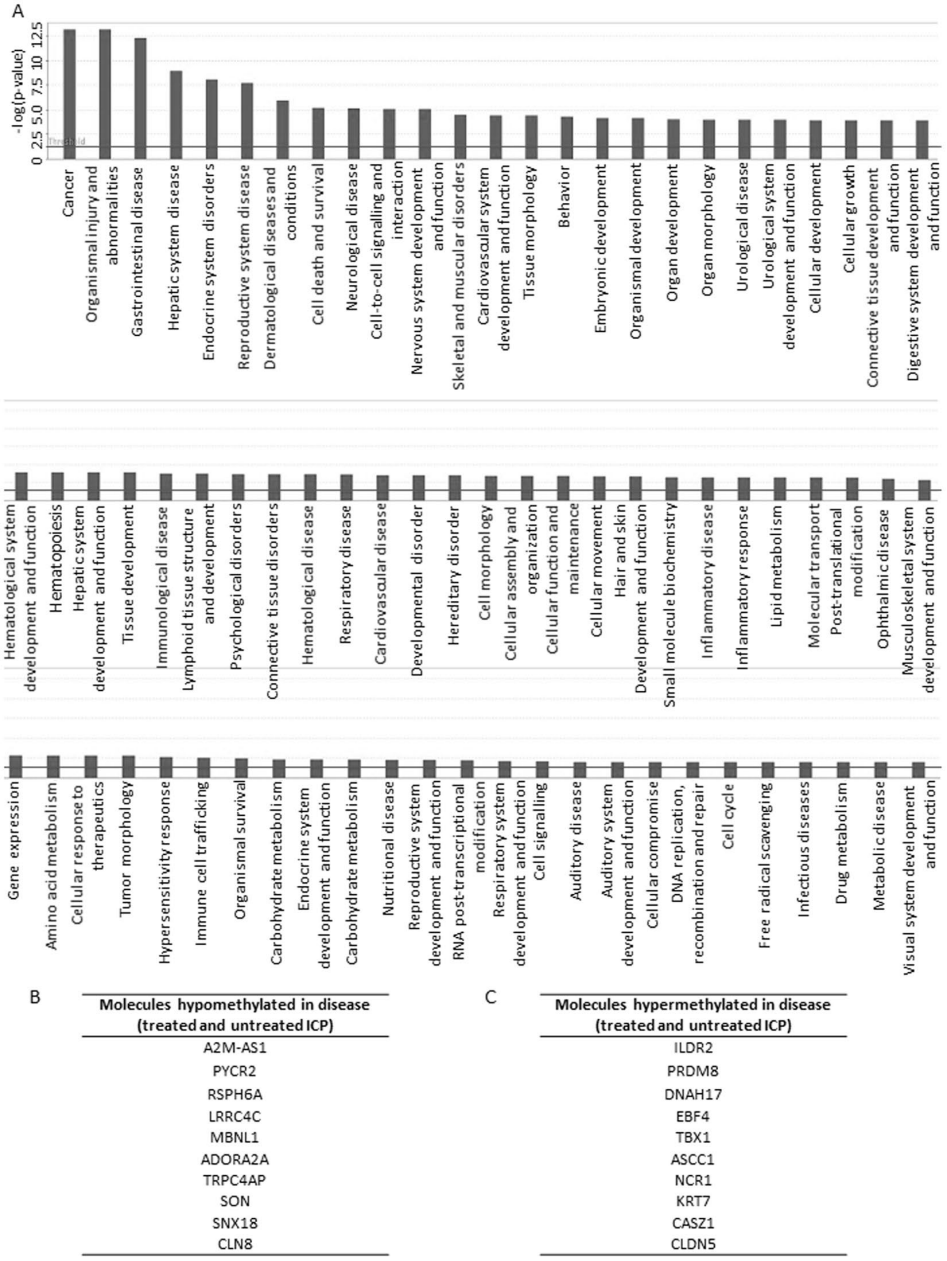
Disease processes and differentially methylated molecules identified by IPA in the control versus disease comparison (treated and untreated ICP). (**A**) Biological processes. Bars represent –log (p value). Continuous line represents p value threshold equivalent to 0.05. (**B**) Molecules Hypomethylated in disease (**C**) Molecules hypermethylated in disease.

To further analyse the biological importance of the methylation differences in each comparison, canonical pathways were identified using IPA, GO and KEGG tools through the MissMethyl R package. In the control versus disease analysis, IPA identified 45 pathways that were differentially methylated, including one related to fatty acid oxidation (Fig. 7). Additional pathways were identified with the R gometh function in the MissMethyl package by GO and KEGG (Table 2). IPA analysis, using the Ingenuity knowledge base reference gene set, also highlighted the presence of gene promoters that were differentially methylated between controls and disease (Fig. 8B). Similarly, several disease processes were revealed for the same comparison, including endocrine system disorders, lipid metabolism, carbohydrate metabolism and metabolic disease (Fig. 8A).

**Table 2:** Canonical pathways identified in the control versus disease comparison (treated and untreated ICP). Canonical pathways were identified using GO and KEGG tools through the MissMethyl R package. n = number of genes.

	Pathway	n	p value
Kyoto Encyclopedia of Genes and Genomes (KEGG)	Pentose phosphate pathway	30	0.018765
	Arginine and proline metabolism	49	0.021207
	Inflammatory bowel disease (IBD)	63	0.03475
	Biosynthesis of amino acids	73	0.037643
	Carbon metabolism	116	0.063953
	Th17 cell differentiation	105	0.081436
	Jak-STAT signaling pathway	152	0.089217
	Cytokine-cytokine receptor interaction	261	0.098618
	Metabolic pathways	1255	0.175827
	Cellular senescence	157	1
Gene ontology (GO)	interleukin-21 receptor activity	1	0.00058
	pyrroline-5-carboxylate reductase activity	3	0.000959
	glucose 1-dehydrogenase [NAD(P)] activity	1	0.001479
	glucose dehydrogenase activity	1	0.001479
	6-phosphogluconolactonase activity	2	0.001617
	regulation of histone H2B conserved C-terminal lysine ubiquitination	1	0.001656
	regulation of DNA double-strand break processing	2	0.001787
	L-proline biosynthetic process	5	0.001903
	proline biosynthetic process	5	0.001903
	positive regulation of skeletal muscle fiber differentiation	2	0.002738
	positive regulation of tongue muscle cell differentiation	2	0.002738
	regulation of tongue muscle cell differentiation	2	0.002738
	tongue muscle cell differentiation	2	0.002738
	glucose-6-phosphate dehydrogenase activity	2	0.002793
	histone H2B conserved C-terminal lysine ubiquitination	2	0.003224
	vagus nerve morphogenesis	2	0.003423
	regulation of histone H2B ubiquitination	5	0.003607
	proline metabolic process	9	0.00394
	regulation of skeletal muscle fiber differentiation	3	0.004158
	skeletal muscle fiber differentiation	5	0.004653

### ICP affects the methylation profile of human placentas

**Figure 9:**
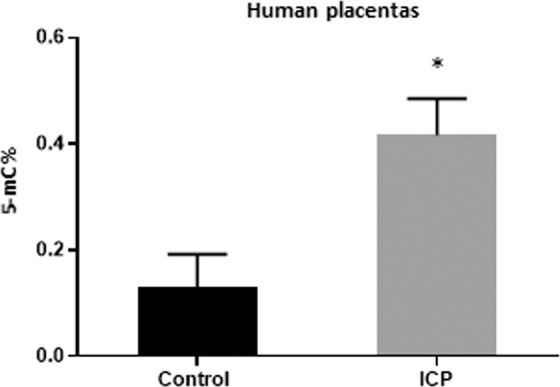
Global DNA methylation patterns in placentas from pregnancies affected by ICP and controls. Data are presented as mean and standard error of the mean (sem). 5-mC%: percentage of 5-methylcytosie; ICP: Intrahepatic cholestasis of pregnancy. *p < 0.05.

The placenta plays an important role in transferring maternal environmental cues to the fetus, thereby potentially influencing metabolic programming^35^. Placentas from ICP pregnancies had a significant increase in global methylation when compared to placentas from uncomplicated pregnancies (Fig. 9).

## Discussion

In this study we demonstrated for the first time that UDCA has a protective role against the development of ICP-associated fetal dyslipidemia. If persistent, these effects may protect offspring from the development of metabolic disease in later life. Using a model of mouse gestational hypercholanemia that was also administered UDCA, we showed a similar effect in the mother and fetus. In addition, UDCA prevented the development of glucose intolerance in the obese offspring of these mice. We also showed for the first time that ICP induces epigenomic alterations in fetal cord blood. Similarly, maternal hypercholanemia altered global methylation patterns in human placentas.

A limited number of studies have assessed effects of UDCA on the fetoplacental unit and whether it may have a beneficial role for the long-term health of the offspring. One previous study revealed raised numbers of syncytial knots in ICP placentas, indicating that elevated BA concentrations in ICP are associated with increased apoptosis^36^. These observations were consistent with a study in a rat model of gestational hypercholanemia that demonstrated increased apoptosis and oxidative stress in placentas exposed to excessive BA^37^. Another study reported a UDCA-induced reversal of transient latent cholestasis in 21-day old offspring from a rat model of hypercholanemic pregnancy^38^. In ICP, UDCA has also been shown to reduce BA levels in umbilical cord blood and amniotic fluid^39,40^.

While in our mouse model UDCA successfully reversed maternal gestational hypercholanemia, it did not reverse increased BA concentrations in the fetal serum. However, we showed UDCA-associated induction of fetal hepatic BA transporters, and this may reduce fetal BA toxicity. It is likely that the upregulation of Mrp2 and Oatp1a1 in the fetal liver protects the fetus from hepatocellular accumulation of potentially toxic biliary constituents. In support of this, an increase of Mrp2 mRNA has been reported in rat cholestasis^41^.

Interestingly, we not only showed that UDCA improved fetal and maternal dyslipidemia in our animal model, but also that it had an impact on FFA synthesis pathways in the fetus. Unlike in the maternal liver, fetal hepatic Fas mRNA expression returned to baseline when hypercholanemic mothers were administered UDCA. This finding is of interest as FFA accumulation in the hepatocyte has been implicated in the development of hepatosteatosis^33^, which is one of the long-term effects of maternal hypercholanemia in the offspring of this model^21^. Emerging evidence suggests that hepatosteatosis precedes the metabolic syndrome and is associated with insulin resistance^42^. We previously established in this model that mild hepatosteatosis precedes progression of glucose intolerance that is unmasked when the offspring are fed an obesogenic diet. Herein, we report that WD-fed female offspring exposed to UDCA *in utero* were protected from impaired glucose tolerance.

This concurs with previous studies in rodents that have successfully improved obesity-induced hepatosteatosis with UDCA administration^43,44^. A permanent effect of UDCA on the pathogenesis of non-alcoholic fatty liver disease (NAFLD) and associated impairment of metabolic health is therefore plausible, and might be attributable to the restoration of a healthy FFA metabolism.

UDCA reduced hepatic TG in hypercholanemic mothers, as well as in the fetal serum and placenta. It also reversed the hypercholesterolemic phenotype of the mother and placentas, and the CA-induced reduction in HDL levels in the fetal serum. Curiously, these results were not accompanied by a reduction in mRNA expression of maternal and fetal hepatic cholesterol *de novo* biosynthesis gene Hmgcr, suggesting that UDCA mediates these effects via alternative mechanisms.

DNA methylation is a typical chromatin modification that can occur when the mother is exposed to an environmental stimulus. We previously established that female offspring of the *Avy* mouse model, when born to mothers fed a CA diet, have reduced methylation of the *IAP* promoter^21^, suggesting that maternal hypercholanemia can trigger epigenetic changes in the offspring. By performing an EWAS in fetal umbilical cord blood, we found that ICP also induces methylation changes in pathways that could contribute to the offspring phenotype. When ICP cases (both treated and untreated) were compared with controls (uncomplicated pregnancies), a total of eighteen differentially methylated CpGs were found. IPA pathway analysis showed that these methylated CpGs are involved in fatty acid oxidation pathways, as well as pathways potentially related to glucose metabolism such as glucose-6-phosphate dehydrogenase activity and glucose 1-dehydrogenase [NAD(P)] activity. Interestingly, such pathways are involved in diseases related to endocrine system, cardiovascular disease, lipid metabolism, carbohydrate metabolism, and metabolic disease, all of which are consistent with our previous findings.

The control versus untreated and the treated versus untreated comparisons yielded limited results and did not identify any loci directly relevant to the phenotype studied. The relatively small number of samples precluded detailed investigation of whether beneficial effects of UDCA stem from epigenetic changes or not. Another limitation of the analysis is that the samples from UDCA-treated and untreated ICP cases were not matched for disease severity as they would be in a randomised, placebo-controlled trial.

While the use of surrogate tissues for human EWAS offers valuable insights into the epigenome, it also presents a challenge for interpretation due to possible tissue-specific epigenetic alterations. However, alterations in DNA methylation in the placenta have been previously reported^36^, suggesting that placental epigenetic marks may be of relevance to metabolic homeostasis in later life.

To gain further insight into the epigenetic component of hypercholenemic pregnancy, we also performed a global DNA methylation analysis of human placentas. As suggested by the EWAS results, ICP altered the human placental epigenome.

## Conclusion

UDCA treatment improves ICP-associated fetal dyslipidaemia. This finding is consistent in mouse hypercholanemic pregnancy. In addition, maternal UDCA administration improved the lipid profile of mothers and placentas and triggered hepatoprotective mechanisms in the fetal liver in mouse gestational hypercholanemia. In adult offspring, UDCA prevented the development of glucose intolerance. Our data support an epigenetic component to the impact of ICP upon offspring metabolic phenotypes. Larger sample sizes will enable more detailed assessment of the effects of UDCA on the epigenome, and investigation of sex-specific alterations. Human cohort studies will be necessary to investigate whether such UDCA effects can persist through adulthood. These data indicate that UDCA may be used as an effective intervention in ICP to prevent susceptibility of the adult offspring to metabolic disease.

## Supplementary information

Supplementary information.
