# Supplementary material for: Ursodeoxycholic acid improves feto-placental and offspring metabolic outcomes in hypercholanemic pregnancy
Source: Sci Rep. 2020 Jun 25;10:10361. doi: 10.1038/s41598-020-67301-1 (PMC7316783; doi:10.1038/s41598-020-67301-1)
Supplement: Supplementary file 1 — Supplementary information. [file 41598_2020_67301_MOESM1_ESM.pdf]

Ursodeoxycholic acid improves feto-placental and offspring metabolic outcomes in hypercholanemic pregnancy

Luiza Borges Manna*[1], Georgia Papacleovoulou*[1], Flavia Flaviani[1,2], Vanessa Formigo-Pataia[1], Asaad Qadri[1], Shadi Abu-Hayyeh[1], Saraid Mcilvride[1], Eugene Jansen[3], Peter Dixon[1], Jennifer Chambers[4], Marta Vazquez Lopez[4], Annika Wahlstroem[5], Negusse Kitaba[6,7], Hanns-Ulrich Marschall[5], Keith M Godfrey[6], Karen Lilycrop[6,7] and Catherine Williamson[1]

1. Division of Women and Children's Health, King's College London, London, United Kingdom 2. NIHR Biomedical Research Centre at Guy's and St Thomas' Foundation Trust, London, United Kingdom 3.Centre for Health Protection, National Institute for Public Health and the Environment, Bilthoven, The Netherlands 4.Women's Health Research Centre, Surgery and Cancer, Faculty of Medicine, Hammersmith Hospital, Imperial College London, London, United Kingdom 5. Department of Molecular and Clinical Medicine/Wallenberg Laboratory, Sahlgrenska Academy, University of Gothenburg, Gothenburg, Sweden 6. MRC Lifecourse Epidemiology Unit and NIHR Southampton Biomedical Research Centre, University of Southampton and University Hospital Southampton NHS Foundation Trust, Southampton, United Kingdom 7. Biological Sciences, University of Southampton, Southampton, United Kingdom. * These authors contributed equally to this work

Gene	Primer sequences
**Control**	
**Cyclophilin**	Forward TGGAGAGCACCAAGACAGACA
	Reverse TGCCGGAGTCGACAATGAT
**FXR targets**	
**Bsep**	Forward AAGCTACATCTGCCTTAGACACAGAA
	Reverse CAATACAGTCCGACCCTCTCT
**Cyp7a1**	Forward AGCAACTAAACAACCTGCCAGTACTA
	Reverse GTCCGGATATTCAAGGATGCA
**Shp**	Forward CGATCCTCTTCAACCCAGATG
	Reverse AGGGCTCCAAGACTTCACACA
**BA transport**	
**Mrp2**	Forward CTCGGCCTTGCTTCTGGTTA
	Reverse TGTGTGATGTTGAGGGCGTT
**Mrp3**	Forward GCAGCAGAACCAAGCATCAAG
	Reverse GCACGCATTCCTCAAACCTGG
**Oatp1a1**	Forward TGAGAAAGACAGCAGTAGGACT
	Reverse TCAGAAACACCTTCATCTTGGAGA
**Oatp1b2**	Forward GCCTGAGTTCAGGACACCAA
	Reverse TACCCTATGCCTTCCACCGA
**Lipid synthesis**	
**Fas**	Forward CCCAGAGGCTTGTGCTGACT
	Reverse CGAATGTGCTTGGCTTGGT
**Hmgcr**	Forward GTGACCTTCGATTATGCGATCA
	Reverse AAGTACATTCTGGGTATTGCTG

Supplementary Table 1: Primer sequences. Primer sequences used for quantitative real-time PCR experiments.

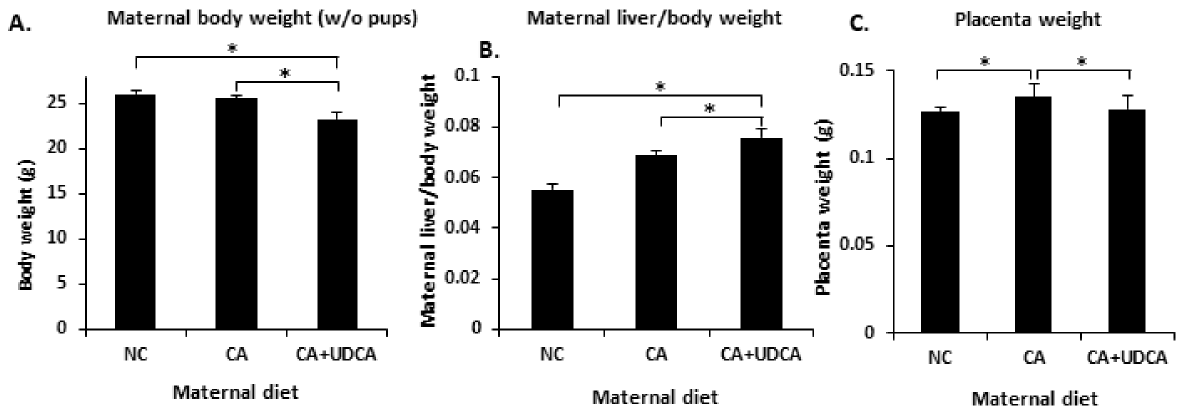

Supplementary Figure 1: Effects of UDCA on mice morphometry according to diet. Effects of UDCA on maternal body weight without pups (A), maternal liver/body weight ratio (B) and placenta weight (C). Data are presented as mean and standard error of the mean (sem) and were analyzed with multiple measures of ANOVA followed by Neuman Keul's post-hoc testing. NC n=6, CA n=6, CA+UDCA n=4. *: $p<0.05$ between comparisons connected by lines. NC: normal chow; CA: diet supplemented with cholic acid; CA+UDCA: diet supplemented with cholic acid + ursodeoxycholic acid.
